# Sarcopenia negatively affects postoperative short-term outcomes of patients with non-cirrhosis liver cancer

**DOI:** 10.1186/s12885-023-10643-6

**Published:** 2023-03-06

**Authors:** Jinhuan Yang, Daojie Wang, Lei Ma, Xuewen An, Zijing Hu, Huili Zhu, Wanqian Zhang, Kaiwen Chen, Jun Ma, Yan Yang, Lijun Wu, Gang Chen, Yi Wang

**Affiliations:** 1 Department of Hepatobiliary Surgery, The First Affiliated Hospital of Wenzhou Medical University, Wenzhou, Zhejiang 325035 China; 2 School of Laboratory Medicine and Life Science, Wenzhou Medical University, Wenzhou, Zhejiang China; 3 School of Nursing, Wenzhou Medical University, Wenzhou, Zhejiang China; 4 The First School of Medicine, School of Information and Engineering, Wenzhou Medical University, Wenzhou, Zhejiang China; 5 Department of Epidemiology and Biostatistics, School of Public Health and Management, Wenzhou Medical University, Chashan High Education Zone, Wenzhou, Zhejiang 325035 China; 6 Key Laboratory of Diagnosis and Treatment of Severe Hepato-Pancreatic Diseases of Zhejiang Province, The First Affiliated Hospital of Wenzhou Medical University, Wenzhou, Zhejiang 325035 China

**Keywords:** Sarcopenia, Non-cirrhosis, Liver cancer, Muscle strength, Short-term outcomes

## Abstract

**Background:**

Literature review have shown that sarcopenia substantially alters the postoperative outcomes after liver resection for malignant tumors. However, these retrospective studies do not distinguish cirrhotic and non-cirrhotic liver cancer patients, nor combine the assessment of muscle strength in addition to muscle mass. The purpose of this study is to study the relationship between sarcopenia and short-term outcomes after hepatectomy in patients with non-cirrhotic liver cancer.

**Methods:**

From December 2020 to October 2021, 431 consecutive inpatients were prospectively enrolled in this study. Muscle strength and mass were assessed by handgrip strength and the skeletal muscle index (SMI) on preoperative computed tomographic scans, respectively. Based on the SMI and the handgrip strength, patients were divided into four groups: group A (low muscle mass and strength), group B (low muscle mass and normal muscle strength), group C (low muscle strength and normal muscle mass), and group D (normal muscle mass and strength). The main outcome was major complications and the secondary outcome was 90-d Readmission rate.

**Results:**

After strictly exclusion, 171 non-cirrhosis patients (median age, 59.00 [IQR, 50.00–67.00] years; 72 females [42.1%]) were selected in the final analysis. Patients in group A had a statistically significantly higher incidence of major postoperative complications (Clavien–Dindo classification ≥ III) (26.1%, *p* = 0.032), blood transfusion rate (65.2%, *p* < 0.001), 90-day readmission rate (21.7%, *p* = 0.037) and hospitalization expenses (60,842.00 [IQR, 35,563.10–87,575.30], *p* < 0.001) than other groups. Sarcopenia (hazard ratio, 4.21; 95% CI, 1.44–9.48; *p* = 0.025) and open approach (hazard ratio, 2.56; 95% CI, 1.01–6.49; *p* = 0.004) were independent risk factors associated with major postoperative complications.

**Conclusions:**

Sarcopenia is closely related to poor short-term postoperative outcomes in non-cirrhosis liver cancer patients and the assessment that combines muscle strength and muscle mass can simply and comprehensively identify it.

**Trial registration:**

ClinicalTrials.gov identifiers NCT04637048. (19/11/2020).

**Supplementary Information:**

The online version contains supplementary material available at 10.1186/s12885-023-10643-6.

## Introduction

Liver cancer is one of the common malignant tumors with poor prognosis. According to the latest report of global cancer statistics, liver cancer is the sixth most commonly diagnosed cancer and the third leading cause of cancer death worldwide in 2020, with approximately 906,000 new cases and 830,000 deaths [1]. Surgical resection is currently the main treatment for liver malignant tumors [2]. With the improvement of surgical technology and perioperative management, the hospitalization stay and the risk of complications (such as postoperative decompensation) after liver resection for malignant tumors are reduced. Nonetheless, the high risk of complications still exists, leading to the delay of adjuvant treatment and increase of hospitalization stay and expenses [3, 4]. Therefore, early preoperative recognition of perioperative risk factors is essential to targeted intervention to correct the possible adverse consequences of these risk factors.

Intraoperative bleeding [5], cirrhosis [6], non-alcoholic fatty liver disease (NAFLD) [7], body mass index (BMI) [8] and pre-operative nutritional status [9] have been previously considered to be factors associated with higher postoperative morbidity or mortality. However, recent studies have found that sarcopenia, a disease of progressive and systemic loss of muscle mass and muscle strength with age [10], could significantly increase the morbidity of post-treatment recurrence and overall complications in patients with liver resection for malignant tumors [11–15]. However, these studies have some common limitations that distinction between cirrhosis liver cancer and non-cirrhosis liver cancer are not taken into account. It is well known that cirrhosis liver cancer patients have lower postoperative platelets, longer postoperative hospital stay and higher perioperative morbidity and mortality than non-cirrhosis liver cancer patients [16]. Therefore, the data of mixed cirrhosis liver cancer and non-cirrhosis liver cancer may cause some selection bias. In addition, most studies are retrospective data and do not combine with the assessment of muscle strength, which is superior to muscle mass in well identifying sarcopenia and predicting adverse consequences [10]. To date, studies that combine muscle strength and muscle mass to research the prognosis of non-cirrhotic liver cancer patients have not been reported. The purpose of this study is to evaluate the effect of sarcopenia on postoperative short-outcomes in patients with non-cirrhotic liver cancer based on the definition of sarcopenia in EWGSOP2 [10].

## Methods

**Fig. 1:**
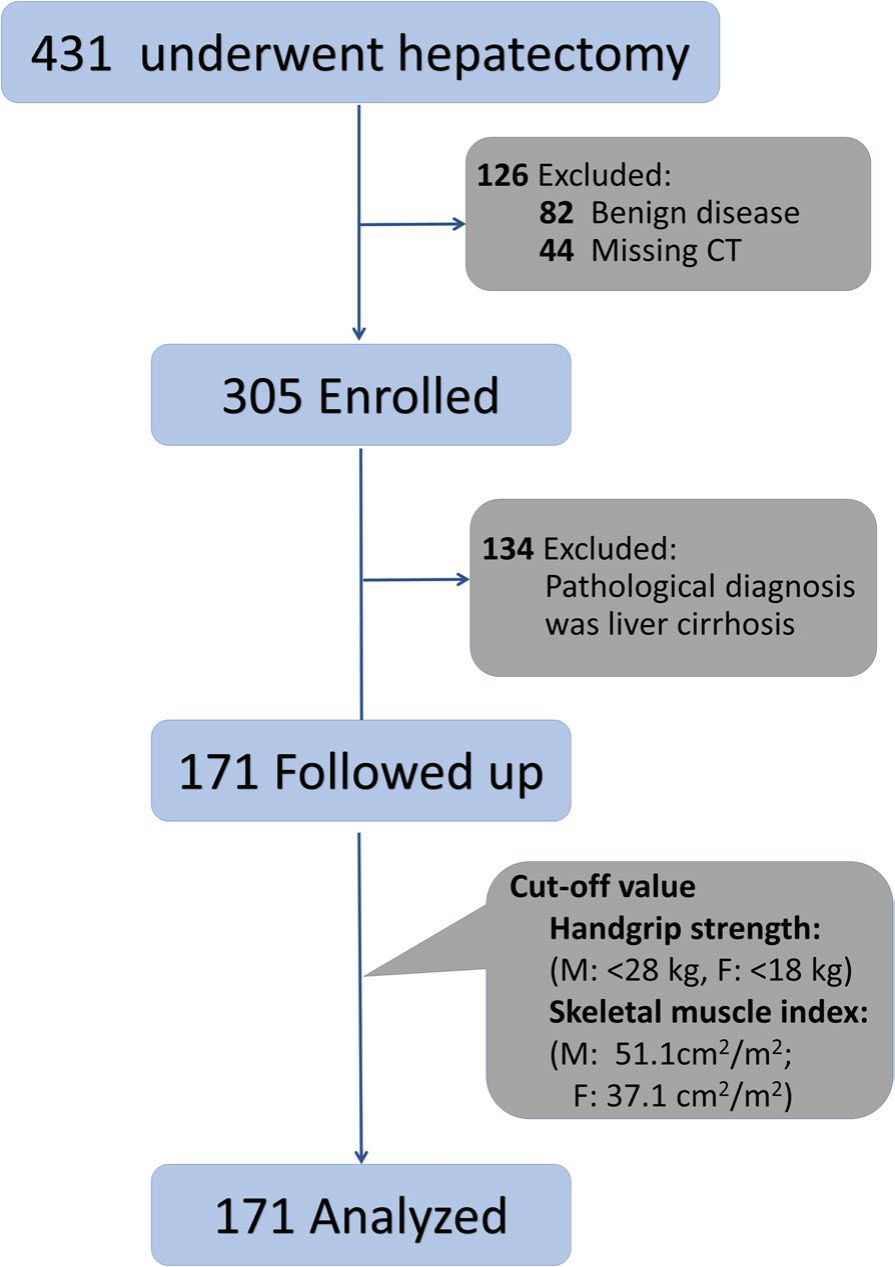
Flow chart of research design

All participants were selected from our prospective database (ClinicalTrials.gov identifiers NCT04637048) from December 2020 to October 2021, among those who underwent hepatobiliary surgery were enrolled for retrospective analysis. The patients received SARC-F questionnaire [17], then the muscle strength (handgrip strength and chair stand test), physical performance (gait speed) and lifestyle questionnaire were recorded. The SMI was calculated based on preoperative computed tomography (CT) scans. In addition, the patient demographics, hematological index for the first time after admission (before taking medication), type of surgical approach, intraoperative data (blood loss and operative time) and short-term postoperative outcomes (blood transfusion, hospital stay, hospitalization expenses, complications, and 90-day readmission) were recorded. The following patients were excluded: (1) Benign disease, (2) Missing CT data or CT scan does not reach L3 level, (3) Pathological diagnosis was liver cirrhosis. Finally, 171 patients were enrolled in the final study (Fig. 1).

The trial protocol (Protocol) conforms to the ethical guidelines of the Declaration of Helsinki [18] and was approved by Review of Ethics Committee in Clinical Research (ECCR) of the First Affiliated Hospital of Wenzhou Medical University, approval number KY2020-074. This study followed the strengthening the reporting of observational studies in epidemiology (STROBE) reporting guideline. The situation of the experiment was explained fully and in detail by researchers, and the written informed consent of each patient was obtained.

### Screening for sarcopenia and analysis of body composition

#### Muscle mass and adipose tissue

**Fig. 2:**
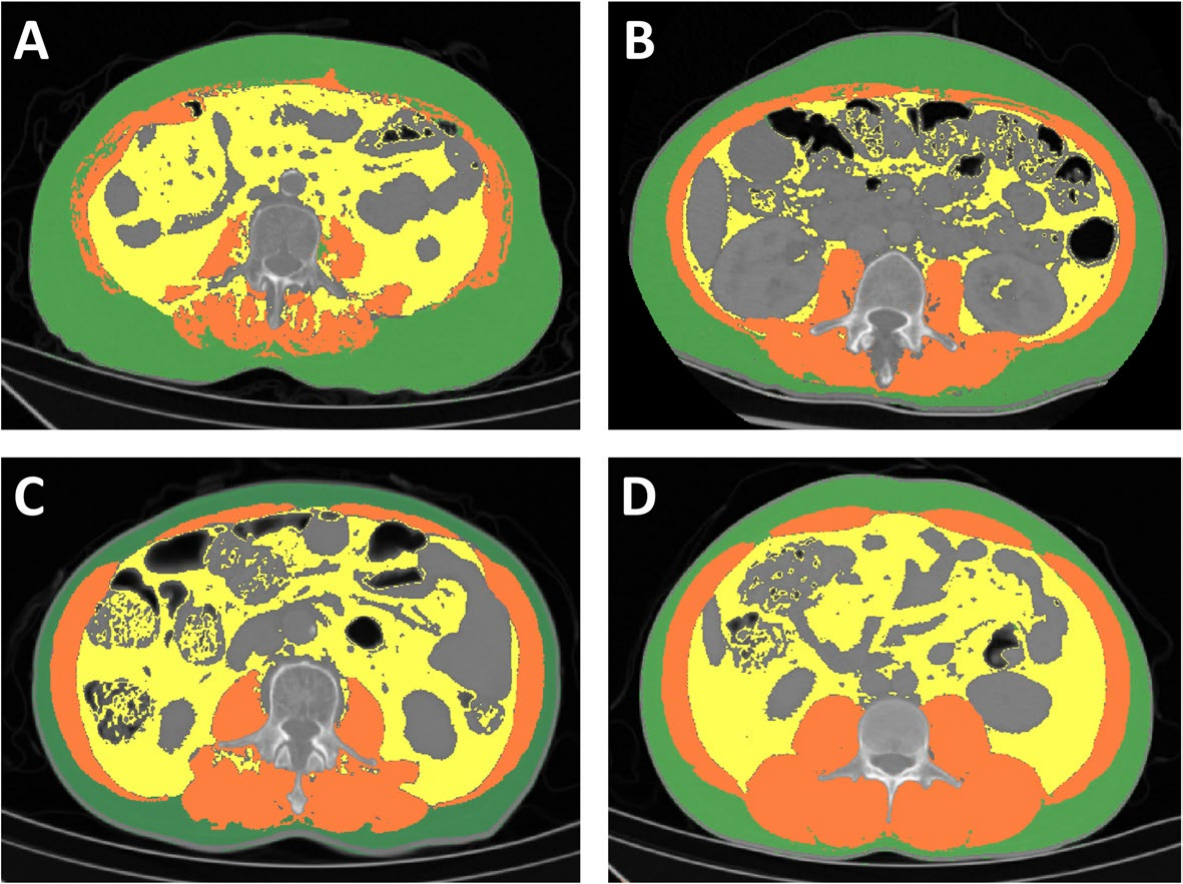
Computed tomographic scans showing areas of skeletal muscle (red), visceral adipose tissue (yellow) and subcutaneous adipose tissue (green) in non-cirrhotic liver cancer patients of the 4 groups (**A-D** corresponds to group A-D)

The CT images of the patients within one week before operation were used to intercept the level of the third (L3) lumbar vertebra [19]. At this level, using Materialise Mimics 21.0 software, tissue Hounsfield unit (HU) thresholds were employed as follows: –29 to + 150 HU for skeletal muscle, -190 to -30 HU for subcutaneous adipose tissue and -150 to -50 HU for visceral adipose tissue (Fig. 2). The skeletal muscle index (SMI) was calculated as the total cross-sectional skeletal muscle area in the L3 plane (cm^2^) / the square of height (m^2^). Male < 51.1cm^2^/m^2^ and female < 37.1 cm^2^/m^2^ were defined as cut-off values of low muscle mass.

#### Muscle strength and physical performance

After admission, the handgrip strength of all patients was tested with a grip meter before treatment. The dominant hand and the non-dominant hand were measured twice intermittently, and an average value of four values was obtained, and the results were averaged (in kilograms). A cutoff of less than 28 kg for men and less than 18 kg for women was used to define patients with low muscle strength [20].

The chair stand test measures the amount of time for patients to rise five times from a seated position without using their arms according to the standard recommended by EWGSOP2 [10].

Gait speed: a route with a length of 8 m was marked and participants were asked to walk along it at their usual speed and the time was recorded.

Finally, based on the SMI and the handgrip strength, all patients were divided into 4 groups: group A (low muscle mass and strength), group B (low muscle mass and normal muscle strength), group C (low muscle strength and normal muscle mass), and group D (normal muscle mass and strength).

### Statistical analysis

The distribution of variables was assessed using the Kolmogorov–Smirnov test and the Shapiro–Wilk test. Data are provided as mean and standard deviation (SD) for continuous variables and as the median (interquartile range [IQR]) for nonparametric distribution of data. Categorical data are expressed as a number (percentage).

χ^2^ test or Fisher’s exact test with Yates correction were used to compare differences in categorical variables. The Kruskal–Wallis test was used for continuous non-parametric variables and analysis of variance was used to compare continuous parameter variables. To estimate the incidence of major postoperative complications, multivariable logistic regression that included statistically significant risk factors in the univariable analysis was performed. A *P*-value < 0.05 was considered statistically significant. All statistical analyses were performed using SPSS software, version 21.0.

## Results

A total of 431 patients were assessed for eligibility in this study. Excluded were 82 patients with benign disease, and 44 patients were excluded because missing CT data or CT scan does not reach L3 level. In addition, 134 patients whose pathological diagnosis was liver cirrhosis were excluded. Finally, 171 patients were enrolled in the final analysis.

The median age of patients was 59.00 (IQR, 50.00–67.00) years, with 99 men (57.9%) and 72 women (42.1%). The median BMI was 22.86 (IQR, 20.94–25.08) kg/m^2^. In terms of parameters related to sarcopenia, the median SMI was 42.22 (IQR, 36.22–49.90) cm^2^/m^2^ and the median handgrip strength was 27.00 (IQR, 20.50–35.00) kg. In addition, the median chair stand test was 14.50 (IQR, 12.00–16.72) s and the median gait speed was 1.00 (IQR, 0.87–1.10) s. As for the short-term postoperative results, a total of 21 patients (12.3%) had major postoperative complications, and the 90-day readmission rate was 7.6% (13 of 171).

### Subgroup analysis according to the presence of sarcopenia

**Fig. 3:**
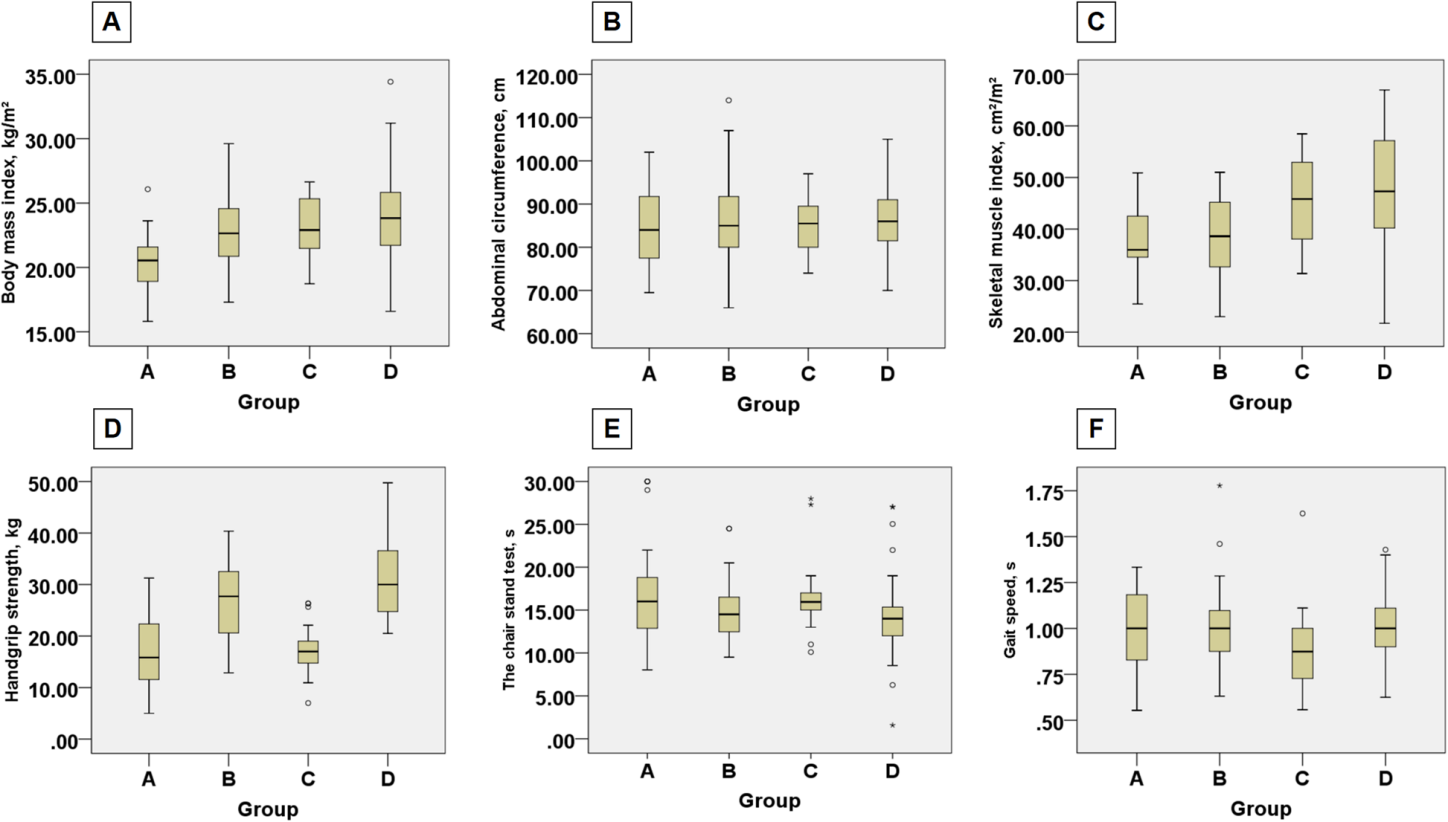
Comparison of indexes reflecting muscle strength, muscle mass and body composition among the four groups. **A-F**, the median (interquartile range) of BMI, abdominal circumference, SMI, handgrip strength, chair stand test and gait speed are shown

Based on the receiver operating characteristic curve analysis, the thresholds of SMI for defining patients with low muscle mass were 51.1 cm^2^/m^2^ for men and 37.1 cm^2^/m^2^ for women. The thresholds of handgrip strength for defining patients with low muscle strength were 28 kg for men and 18 kg for women. All patients were divided into 4 groups based on the SMI and handgrip strength (Table 1). Patients with low muscle strength and muscle mass (group A) had a statistically significantly lower median SMI (35.97 [IQR, 34.53–44.41] cm^2^/m^2^ vs 38.60 [IQR, 32.55–45.41] cm^2^/m^2^ in group B, 45.82 [IQR, 38.00–52.97] cm^2^/m^2^ in group C and 47.30 [IQR, 40.15–57.30] cm^2^/m^2^ in group D, *p* < 0.001), weaker handgrip strength (15.83 [IQR, 11.50–22.50] kg vs 28.00 [IQR, 22.50–34.68] kg in group B, 17.00 [IQR, 14.19–19.78] kg in group C and 33.20[IQR, 25.68–38.05] kg in group D, *p* < 0,001), longer time of the chair stand test (16.00 [IQR,12.75–19.60] s in group A vs 14.50 [IQR, 12.44–16.50] s in group B, 15.95 [IQR, 14.75–17.36] s in group C and 14.00 [IQR, 12.00–15.70] s in group D, *p* < 0.001) and a lower BMI (20.54 [IQR, 18.73–21.88] kg/m^2^ vs 22.65 [IQR, 20.76–24.61] kg/m^2^ in group B, 22.91 [IQR, 21.45–25.34] kg/m^2^ in group C and 23.83 [IQR, 21.72–25.85] kg/m^2^ in group D, *p* < 0.001) compared with patients in other groups (Fig. 3). No differences were seen in gait speed, abdominal circumference, visceral adipose tissue, subcutaneous adipose tissue, Child–Pugh stage, Age-adjusted Charlson comorbidity index (ACCI), the Barcelona Clinic Liver Cancer (BCLC) stage and Model for end-stage liver disease (MELD) score. Supplementary Table S1 showed the preoperative hematological indicators of each group.

**Table 1:** Baseline Characteristics of Patients According to Muscle Mass and Muscle Strength

Variable	Median (IQR)					
	**Total (*N* = 171)**	**Group A (*N* = 23)**	**Group B (*N* = 63)**	**Group C (*N* = 18)**	**Group D (*N* = 67)**	***P* value**
Age, y	59.00(50.00–67.00)	67.00(58.00–75.00)	58.00 (50.00–66.00)	67.00(62.75–74.00)	54.00 (48.00–62.00)	0.379
Sex, No. (%)						
Female	72 (42.1)	7 (30.40)	18 (36.5)	9 (50.0)	38(56.7)	0.053
Male	99 (57.9)	16(69.60)	45 (63.5)	9 (50.0)	29(43.3)	
BMI,Kg/m^2^	22.86(20.94–25.08)	20.54(18.73–21.88)	22.65(20.76–24.61)	22.91(21.45–25.34)	23.83(21.72–25.85)	< 0.001
Abdominal circumference, cm	85.00 (79.00–91.50)	84.00 (77.00–92.00)	85.00 (80.00–92.00)	85.50(79.75–89.63)	86.00 (81.00–91.00)	< 0.001
SARC-F, No. (%)						
> = 4	3 (1.8)	2(8.7)	0 (0)	1 (5.6)	0 (0)	0.016
< 4	168 (98.2)	21 (91.3)	63 (100)	17 (94.4)	67 (100)	
Handgrip strength, kg	27.00(20.50–35.00)	15.83(11.50–22.50)	28.00(22.50–34.68)	17.00(14.19–19.78)	33.20(25.68–38.05)	< 0.001
Chair stand test, s	14.50 (12.00–16.72)	16.00 (12.75–19.60)	14.50 (12.44–16.50)	15.95(14.75–17.36)	14.00(12.00–15.70)	< 0.001
Gait speed, m/s	1.00 (0.87–1.10)	1.00 (0.80–1.20)	1.00 (0.88–1.10)	0.87 (0.72–1.00)	1.00 (0.90–1.11)	0.124
SMI, cm^2^/m^2^	42.22(36.22–49.90)	35.97(34.53–44.41)	38.60(32.55–45.41)	45.82(38.00–52.97)	47.30(40.15–57.30)	< 0.001
Visceral adipose tissue, cm^2^	118.08(103.87–132.58)	108.31(87.98–124.31)	111.77(97.98–130.98)	107.68(80.23–123.41)	127.11(114.57–143.23)	0.249
Subcutaneous adipose tissue, cm^2^	118.35(92.35–130.40)	92.35(64.62–121.47)	105.57(62.34–127.97)	106.87(87.57–121.20)	122.83(114.57–137.95)	0.190
Cause, No. (%)						
Hepatocellular carcinoma	47 (27.5)	8 (34.5)	13 (20.6)	6(33.3)	20 (29.9)	0.714
Colorectal liver metastases	44 (25.7)	4 (17.2)	22 (34.9)	4(22.2)	16 (26.7)	
Cholangiocarcinoma	67 (39.2)	8 (34.5)	24 (38.1)	8(44.4)	25 (37.3)	
Gallbladder cancer	7 (4.1)	1 (4.3)	3 (4.8)	0	3 (4.5)	
Mixed liver cancer	6 (3.5)	2 (8.6)	1 (1.6)	0	3 (4.5)	
ASA grade, No. (%)						
I	8 (4.7)	0 (0)	2 (3.2)	2 (11.1)	4(6.0)	0.152
II	159 (93.0)	21 (91.3)	59 (93.6)	16 (88.9)	63(94.0)	
III	4 (2.3)	2 (8.7)	2 (3.2)	0 (0)	0 (0)	
Ascites, No. (%)	8 (4.7)	2 (8.7)	1(1.6)	3 (16.7)	2(3.0)	0.038
Age-adjusted Charlson comorbidity score	6.00 (5.00–7.00)	6.00 (5.00–6.00)	6.00 (5.00–6.00)	6.00 (5.00–7.00)	6.00 (5.00–7.00)	0.827
Child–Pugh stage, No. (%)						
A	102 (59.6)	13 (56.5)	37(58.7)	11 (61.1)	41 (61.2)	0.997
B	7 (4.1)	1 (4.3)	2 (3.2)	1 (5.6)	3 (4.5)	
MELD score	8.00 (7.00–9.00)	8.00 (7.00–9.00)	8 00 (7.00–8.00)	8.00 (7.00–8.00)	8.00 (7.00–9.00)	0.174
BCLC stage, No. (%)						
0	22 (12.9)	3 (13.0)	8 (12.7)	2 (11.1)	9 (13.4)	0.993
A	87 (50.9)	13 (56.5)	31 (49.2)	8 (44.4)	35 (52.2)	
B	17(10.0)	3 (13.0)	6(9.5)	2(11.1)	6 (9.0)	
C	45 (17.0)	4 (17.4)	18 (28.6)	6 (33.3)	17 (25.4)	
Microvascular invasion, No. (%)	17 (9.9)	2 (8.7)	7 (11.1)	2 (11.1)	6 (9.4)	0.899
Satellite stove, No. (%)	16 (9.68)	2 (8.7)	7 (11.1)	2 (11.1)	5 (7.5)	0.750
Lesion size, cm	4.40 (3.60–6.30)	3.80 (3.50–6.00)	4.60 (3.70–6.50)	4.55 (4.00–7.23)	4.50 (3.90–4.80)	0.358
Lesions, No. (%)						
Solitary	151 (88.3)	20 (87.0)	56 (88.9)	16 (88.9)	59 (88.1)	0.995
Multiple	20 (11.7)	3 (13.0)	7 (11.1)	2 (11.1)	8 (11.9)	
Hypertension, No. (%)	57 (33.3)	8 (34.8)	20 (31.7)	8 (44.4)	21 (31.3)	0.750
Diabetes, No. (%)	29 (17.0)	4 (17.4)	6 (9.5)	5 (27.8)	14 (20.9)	0.194
Smoking, No. (%)	62 (36.3)	11 (47.8)	25 (39.7)	5 (27.8)	21(31.3)	0.405
Drinking, No. (%)	50 (29.2)	9 (39.1)	20 (31.7)	5 (27.8)	16(23.9)	0.527
Physical activity, No. (%)						
> 4 h/wk	104 (60.8)	11 (47.8)	40 (63.5)	13(72.2)	40(59.7)	0.418
< = 4 h/wk	67 (39.2)	12 (52.2)	23 (36.5)	5 (27.8)	27(40.3)	
Dietary structure, No. (%)						
Semi-vegetarian	128 (74.8)	14 (60.9)	47 (74.6)	13 (72.2)	54 (80.6)	0.646
Meat eater	14 (8.2)	3 (13.0)	6 (9.5)	1 (5.6)	4 (6.0)	
Vegetarian	29 (17.0)	6 (26.1)	10 (15.9)	4 (22.2)	9 (13.4)	
Sleep time, No. (%)						
> = 8 h	76 (44.4)	12 (52.2)	24 (38.1)	10(55.6)	30(44.8)	0.477
< 8 h	95 (55.6)	11 (47.8)	39 (61.9)	8(44.4)	37(55.2)	

*Abbreviations: Group A* low muscle mass and strength, *Group B* low muscle mass and normal muscle strength, *Group C* low muscle strength and normal muscle mass, *Group D* normal muscle mass and strength, *ASA* American Society of Anesthesiologists, *BMI* body mass index (calculated as weight in kilograms divided by height in meters squared), *MELD* model for end-stage liver disease, *IQR*: interquartile range

Regarding postoperative results (Table 2), a total of 86 patients (50.3%) underwent laparoscopic liver resection, while 61 (35.7%) underwent major hepatectomy. Group A had a statistically significantly higher rate of major postoperative complications (26.1% [6 of 23] vs12.7% [8 of 63] in group B, 22.2% [4 of 18] in group C and 4.5% [3 of 67] in group D, *p* = 0.032) and higher rate of blood transfusion (65.2% [15 of 23] vs 38.1% [24 of 63] in group B, 38.9% [7 of 18] in group C and 23.9% [16 of 67] in group D, *p* ＜0.001) compared with patients in the other groups. In addition, the 90-day readmission rate was higher in group A (21.7% [5 of 23] vs 4.8% [3 of 63] in group B, 11.1% [2 of 18] in group C and 4.5% [3 of 67] in group D, *p* =0.037) as well longer median hospital stay (*p* < 0.001) and higher hospitalization expenses (*p* < 0.001). There was largely no statistical difference in postoperative hematological indicators among the groups (Supplementary Table S2).

**Table 2:** Perioperative Results of Patients According to Muscle Mass and Muscle Strength

	**Median (IQR)**					
**Variable**	**Total (*N* = 171)**	**Group A (*N* = 23)**	**Group B (*N* = 63)**	**Group C (*N* = 18)**	**Group D (*N* = 67)**	***P* value**
Operation mode, No. (%)						
Laparoscopic	86(50.3)	14(60.9)	33(52.4)	12(66.7)	27(40.3)	0.125
Open	85(49.7)	9(39.1)	30(47.6)	6(33.3)	40(59.7)	
Type of hepatectomy, No. (%)						
Major	61 (35.7)	8 (34.8)	26(41.3)	6 (33.3)	21 (31.3)	0.692
Minor	110 (64.3)	15(65.2)	37(58.7)	12 (66.7)	46 ( 68.7)	
Operative time, min	174.00(135.00–200.00)	194.00(175.00–230.00)	170.00(132.00–200.00)	155.00(125.00–192.00)	173.00(132.00–200.00)	0.454
Blood loss, median (ml)						
< = 400	138 (80.7)	16(69.6)	51 (81.0)	15(83.3)	56(83.6)	0.518
> 400	33(19.3)	7(30.4)	12(19.0)	3(16.7)	11 (16.4)	
Clavien-Dindo classification, No. (%)						
I-II	27 (15.8)	5 (21.7)	12 (19.0)	3 (16.7)	7 (10.4)	0.032
III-V	21 (12.3)	6 (26.1)	8 (12.7)	4 (22.2)	3 (4.5)	
Blood Transfusion, No. (%)	62(36.3)	15(65.2)	24(38.1)	7 (38.9)	16 (23.9)	< 0.001
Hospital stay, d	10.00(8.0–14.00)	13.00(10.00–19.00)	10.00(8.00–13.00)	15.00(9.00–21.50)	10.00(7.00–12.00)	< 0.001
Hospitalization expenses	47,594.00(37,757.00–63,580.70)	60,842.00(35,563.10–87,575.30)	47,603.00(39,187.80–61,624.00)	54,557.40(38,840.10–79,014.00)	42,343.50 (35,299.70–57,007.40)	< 0.001
90-d Readmission rate, No. (%)	13(7.6)	5(21.7)	3(4.8)	2 (11.1)	3 (4.5)	0.037

### Risk factors associated with major postoperative complications

Sarcopenia, operation mode and triacylglycerol (TG) were statistically significantly associated with major postoperative complication in the univariable analysis. In the multivariable analysis, sarcopenia (hazard ratio, 4.21; 95% CI, 1.44–9.48; *p* = 0.025) and operation mode (hazard ratio, 2.56; 95% CI, 1.01–6.49; *p* = 0.004) were independent risk factors associated with major postoperative complications (Supplementary Table S3).

## Discussion

This study is the first cohort study to distinguish between non-cirrhosis liver cancer and cirrhosis liver cancer, which reports that sarcopenia will affect the short-term outcomes of non-cirrhosis liver cancer patients after liver resection. Sarcopenia is not only common in cancer patients, but also frequently appears in patients with liver cirrhosis [21]. Liver cirrhosis can cause sarcopenia through many factors, including malnutrition, synthesis dysfunction, hyperammonemia, systemic inflammation, intestinal metabolic disorder and intestinal permeability change [22]. Moreover, liver cirrhosis is one of the risk factors for liver cancer [23]. Liver cirrhosis is formed after a long-term inflammation that results in replacement of the healthy liver parenchyma with fibrotic tissue and regenerative nodules, thus leading to portal hypertension [23]. These changes in liver parenchyma make patients with cirrhosis have a significantly higher risk of postoperative complications and a higher mortality rate than patients without cirrhosis [6, 24]. Therefore, even if the patients with cirrhosis are balanced between groups, mixing patients with and without cirrhosis may cause bias. Our study excluded cirrhosis liver cancer patients, which further proved the role of sarcopenia as an independent factor in the short-term outcomes after liver resection for malignant tumors.

Small-scale studies are limited by retrospective design and the lack of standardized measurements of sarcopenia (assess muscle mass only) [11, 25], thus restricting applicability in clinic. In the latest definition of sarcopenia, EWGSOP2 uses low muscle strength as the primary parameter of sarcopenia. Specifically, sarcopenia is probable when low muscle strength is detected, and it is confirmed by presence of low muscle mass. Further, when low physical performance are detected, sarcopenia is considered severe [10]. The handgrip strength and the chair stand test are recommended to represent muscle strength. A large prospective study shows that weak handgrip strength is related to the increased risk of all-cause death and cardiovascular death [26], and the handgrip strength shows high accuracy in identifying muscle strength decrease and predicting adverse short-term outcomes after liver resection for malignant tumors in our cohort. Studies have also shown that the chair stand test, as a representative of lower limb strength, is closely related to disability and mortality [27, 28]. In this cohort, the results of the chair stand tests are also significantly different between groups, which is consistent with the results of handgrip strength. After admission, the patients were investigated by SARC-F questionnaire. Then, the handgrip strength test and the chair stand test were used to identify low muscle strength and abdominal CT was used to confirm low muscle mass. These assessments can be easily completed in clinical practice and conduce to early detection of sarcopenia. By comprehensively assessing muscle strength and muscle mass, in our cohort, patients with low muscle strength and muscle mass had worse short-term outcomes than patients with low muscle strength only or low muscle mass only.

Many risk factors are unchangeable, such as age, operation mode, intraoperative bleeding [5], liver cirrhosis [6]. Consequently, the factors that can be changed or improved before operation is essential to be focused on. Our study found that sarcopenia was associated with poor short-term outcomes after liver resection for malignant tumors in non-cirrhosis liver cancer patients. If early intervention can be performed before operation, it is likely to improve the short-term outcomes after liver resection for malignant tumors. Published data suggest that improving gut microbiota, dietary supplementation and exercise could alter short-term outcomes after cancer surgery by improving muscle mass and muscle strength (specifically, taking a prebiotic formulation for 13 weeks, consuming sufficient protein [1.2–1.5 g/kg body weight/day] for 12 weeks and do aerobic exercise for at least 20 to 30 min three times a week) [29–32]. However, the long time required for these measures will delay the surgical intervention, thus affecting its clinical applicability because delaying the surgical intervention in patients with liver cancer may make the disease progress and the prognosis worsen. Studies have shown that intravenous injection of iron into mice can improve the handgrip strength of mice within 24 h, and injection of ferric carboxymaltose (a clinically approved supplement) into cancer patients with muscle weakness can improve the handgrip strength (the median handgrip strength of dominant hands increased by 8% and the median handgrip strength of the non-dominant hand increased by 6%) [33]. Early 7-day supplemental parenteral nutrition improves muscle strength in cancer patients, and the median handgrip strength increased by 11.2% after seven days [34]. Treatment with the insulin-sensitizing thiazolidinedione drug rosiglitazone for a week leads to an improvement in muscle mass through inhibiting the decrease of muscle protein synthesis [35]. The above measures might enhance the muscle strength of patients in a short period of time without delaying operation, and contribute to improving sarcopenia—an adverse factor related to short-term postoperative outcomes.

In addition to sarcopenia, body composition, such as adipose tissue, can also be involved in the prognosis of patients with liver cancer. Many studies have reported that visceral adipose tissue is a major contributor to metabolic risk, whereas subcutaneous adipose tissue may have a protective role [36]. Obesity with visceral fat accumulation, which increases the levels of leptin, TNFα, IL-6 and resistin, and decreases the level of adiponectin, is related to cancer risk and mortality [37, 38]. On the contrary, subcutaneous fat-dominant obesity is considered to be a possible “metabolically healthy” status, probably because subcutaneous fat effectively stores excess lipids and subcutaneous adipocytes can exert a favorable metabolic function [39, 40]. In this cohort, no difference were seen in visceral adipose tissue and subcutaneous adipose tissue and they were not independent risk factors associated with major postoperative complications, which may associated with low BMI. The median BMI of patients with sarcopenia in our cohort was 20.54 (IQR, 18.73–21.88) kg/m^2^, which was close to that reported in the Asian population (the BMI was 21.5 ± 3.0 kg/m^2^) [41] while far lower than that reported by the western population (the median BMI was 25.06 (IQR, 23.40–31.18) kg/m^2^) [13]. It may be related to the low incidence of sarcopenic obesity in Asia [20, 42].

With the increasing understanding of liver anatomy and the progress of medical equipment, laparoscopic liver resection (LLR) increased in frequency and complexity. Many studies have shown that LLR and minor hepatectomy have less intraoperative bleeding, fewer postoperative complications and shorter hospital stay compared with open liver resection (OLR) and major hepatectomy [43–45]. In order to avoid bias, we compared them in this study, and found that each group was balanced (operation mode [*p* = 0.125] and type of hepatectomy [*p* = 0.692]). As we all know, Child–Pugh stage is an important index to assess patients’ liver function reserve, and it can be used as a predictor of postoperative outcomes in patients with liver cancer [46]. ACCI [47], BCLC [48] and MLED [49] are all related to the postoperative prognosis of cancer patients. However, in this cohort, there was no difference in Child–Pugh stage(*p* = 0.997), ACCI (*p* = 0.827), BCLC (*p* = 0.993) and MLED (*p* = 0.174) among the groups. The above indexes may not be as good as sarcopenia in predicting major postoperative complications of non-cirrhosis liver cancer patients. In a word, sarcopenia is a powerful index of short-term outcomes after liver resection for malignant tumors.

This cohort study firstly assessed the short-term outcomes after liver resection for malignant tumors of non-cirrhosis liver cancer patients by comprehensively assessing muscle strength and muscle mass, thus avoiding the selection bias caused by simultaneous presence of cirrhosis and noncirrhosis [13]. The factors affecting the postoperative outcomes are balanced among all groups in this cohort, which can better indicate that sarcopenia is an independent risk factor for short-term postoperative outcomes of liver cancer patients without cirrhosis. It is necessary to conduct randomized controlled trials in the future and carry out appropriate intervention on patients based on accurate identification of sarcopenia to find effective measures that can improve the outcomes after liver resection for malignant tumors.

## Conclusions

The analysis of our prospectively kept database indicated the association of sarcopenia with postoperative short-term outcomes of patients with non-cirrhosis liver cancer, shows that the comprehensive assessment of muscle strength and muscle mass is expected to accurately identify sarcopenia clinically and possible intervention will be carried out before operation to improve the postoperative outcomes.

## Supplementary Information

**Additional file 1: Table_S1.** Hematological indicators of the first admission among the groups. **Table_S2.** Postoperative hematological indicators among the groups. **Table_S3.** Logistic Regression for Predictive Factors of Postoperative Complications.

## Data Availability

The datasets used and/or analysed during the current study are available from the corresponding author on reasonable request.
