# Supplementary material for: Sarcopenia negatively affects postoperative short-term outcomes of patients with non-cirrhosis liver cancer
Source: BMC Cancer. 2023 Mar 6;23:212. doi: 10.1186/s12885-023-10643-6 (PMC9987146; doi:10.1186/s12885-023-10643-6)
Supplement: Supplementary file 1 — Additional file 1: Table_S1. Hematological indicators of the first admission among the groups. Table_S2. Postoperative hematological indicators among the groups. Table_S3. Logistic Regression for Predictive Factors of Postoperative Complications. [file 12885_2023_10643_MOESM1_ESM.docx]

***Sarcopenia negatively affects postoperative short-term outcomes of patients with non-cirrhosis liver cancer***

Jinhuan Yang et.al

**Table_S1.** Hematological indicators of the first admission among the groups

| **Variable** | **Median (IQR)** | | | | | **P value** |
| --- | --- | --- | --- | --- | --- | --- |
|  | **Total (N=171)** | **Group A (N=23)** | **Group B (N=63)** | **Group C (N=18)** | **Group D (N=67)** |  |
| AST, U/L | 26.00 (20.00-36.00) | 27.00 (21.00-53.00) | 28.00 (21.00-36.00) | 35.00 (25.75-88.25) | 22.00 (18.00-32.00) | <0.001 |
| ALT, U/L | 23.00(15.00-38.00) | 21.00 (16.00-65.00) | 25.00 (17.00-41.00) | 30.50 (16.50-108.25) | 19.00(12.00-32.00) | 0.029 |
| GGT level, U/L | 38.00 (22.00-83.00) | 107.00(49.00-379.00) | 42.00 (27.00-72.00) | 30.00 (19.00-412.75) | 33.00 (18.00-47.00) | <0.001 |
| Cholesterol, mmol/L | 5.03（4.38-5.91） | 4.87（3.69-5.42） | 4.91（4.36-5.63） | 5.71（4.55-7.11） | 5.18（4.47-6.00） | 0.102 |
| TBIL, Umol/L | 7.00 (1.85-12.00) | 12 (9.00-89.00) | 11.00 (7.00-15.00) | 11.50 (8.00-47.25) | 1.57 (1.04-2.04) | <0.001 |
| Cr, Umol/L | 65.00 (57.00-77.00) | 67.00 (54.00-85.00) | 67.00 (54.00-85.00) | 64.50(47.75-72.25) | 62.00 (55.00-75.00) | 0.194 |
| ALB, g/L | 40.40 (37.50-42.70) | 36.30 (33.20-41.10) | 40.50 (37.50-43.30) | 39.65(36.23-41.95) | 41.30 (39.30-42.70) | 0.002 |
| TG, mmol/L | 1.36 (0.96-2.12) | 1.38 (0.83-2.13) | 1.22 (0.89-1.88) | 1.31 (1.11-2.78) | 1.45 (1.04-2.00) | 0.193 |
| HDL, mmol/L | 1.04 (0.85-1.37) | 0.76 (0.52-1.20) | 1.02 (0.83-1.32) | 1.01 (0.82-1.37) | 1.06 (0.92-1.48) | 0.012 |
| LDL, mmol/L | 2.76 (2.24-3.39) | 2.60 (2.00-3.36) | 2.68 (2.28-3.25) | 3.34 (2.07-4.01) | 2.76 (2.30-3.35) | 0.362 |
| LDH, U/L | 200.00 (178.00-223.00) | 218.00 (176.00-229.00) | 200.00 (180.00-220.00) | 217.50 (195.25-232.25) | 194.00 (176.00-225.00) | 0.264 |
| INR | 0.99 (0.95-1.04) | 0.99 (0.95-1.09) | 1.01 (0.96-1.04) | 0.98 (0.92-1.04) | 0.99 (0.95-1.02) | 0.378 |
| PT, s | 13.10(12.70-13.60) | 13.10(12.70-13.90) | 13.20(12.80-13.60) | 12.85(12.40-13.58) | 13.00(12.70-13.50) | 0.353 |
| AFP, ng/mL | 3.30 (2.20-5.68) | 4.00(2.00-9.91) | 3.90(2.28-8.74) | 2.88(1.85-6.50) | 3.10(2.20-4.50) | 0.339 |
| CEA, Ug/L | 1.80 (1.10-2.90) | 2.00(1.00-3.30) | 1.70(1.10-3.00) | 1.90(1.45-2.63) | 1.60(1.00-2.70) | 0.716 |
| CA199, U/mL | 9.60 (4.70-23.20) | 36.80(9.30-373.20) | 9.10(5.40-26.40) | 16.00(9.00-179.63) | 8.00(3.50-16.40) | 0.001 |
| WBC level, ×10^9^/L | 6.18 (4.88-7.34) | 6.93(5.12-7.76) | 6.15(4.65-6.88) | 5.49(4.25-7.18) | 6.18(5.03-7.74) | 0.148 |
| RBC level, ×10^12^/L | 4.42（4.09-4.76） | 4.17(3.60-4.44) | 4.60(4.25-4.94) | 4.28(3.98-4.50) | 4.43(4.09-4.75) | <0.001 |
| NEUT level, ×10^9^/L | 3.69 (2.80-4.76) | 4.76(3.26-5.78) | 3.62(2.79-4.42) | 3.66(2.34-4.66) | 3.93(2.77-4.88) | 0.129 |
| Monocyte level, ×10^9^/L | 0.43 (0.32-0.55) | 0.43(-.37-0.80) | 0.44(0.32-0.55) | 0.37(0.23-0.55) | 0.41(0.32-0.55) | 0.243 |
| HGB level, g/L | 134.00 (123.00-145.00) | 127.00(114.00-136.00) | 141.00(129.00-154.00) | 128.00(119.50-134.25) | 134.00(126.00-144.00) | 0.002 |
| PLT count, ×10^9^/L | 224.00 (174.00-266.00) | 212.00(173.00-363.00) | 212.00(151.00-260.00) | 252.50(166.00-303.25) | 227.00(192.00-260.00) | 0.266 |
| Fib level, g/L | 2.74 (2.38-3.18) | 3.96(2.79-5.19) | 2.82(2.46-3.34) | 3.25(2.85-4.69) | 2.95(2.59-3.51) | 0.005 |

Abbreviations: ALT, alanine aminotransferase; AST, aspartate aminotransferase; GGT, γ-glutamyltransferase; TBIL, total bilirubin; Cr, creatinine; ALB, albumin; TG, triglyceride; HDL, high density lipoprotein; LDL, low density lipoprotein; LDH, Lactate dehydrogenase; INR, international normalized ratio; PT, Prothrombin time; AFP, alpha-fetoprotein; CEA, carcinoembryonic antigen; CA199, Saccharide antigen 199; WBC, white blood cell; RBC, red blood cell; NEUT, neutrophil; HGB, hemoglobin;PLT, blood platelet; Fib, Fibrinogen.

**Table_S2.** Postoperative hematological indicators among the groups

| **Variable** | **Median (IQR)** | | | | |  |
| --- | --- | --- | --- | --- | --- | --- |
|  | **Total (N=171)** | **Group A (N=23)** | **Group B (N=63)** | **Group C (N=18)** | **Group D (N=67)** | **P value** |
| Hematological indicators on the first day after operation | | | | | | |
| ALT, U/L | 179.00（110.00-370.00） | 218.00(120.00-328.00) | 205.00(115.00-489.00) | 213.50(121.75-405.75) | 156.00(96.00-275.00) | 0.227 |
| AST, U/L | 197.00（116.00-417.00） | 311.00(126.00-449.00) | 240.00(134.00-456.00) | 226.00(124.00-579.00) | 151.00(95.00-292.00) | 0.058 |
| INR | 1.12（1.05-1.17） | 1.15(1.06-1.20) | 1.13(1.08-1.17) | 1.08(0.99-1.18) | 1.10(1.04-1.25) | 0.143 |
| PT, s | 14.50（13.70-15.00） | 14.50（13.60-15.00） | 14.50（14.00-14.90） | 14.25（12.85-15.03） | 14.30（13.50-14.90） | 0.373 |
| WBC level, ×10^9^/L | 11.86（9.30-14.33） | 12.11(9.48-13.90) | 10.82(8.78-13.93) | 12.17(10.14-13.71) | 12.55(9.56-15.62) | 0.333 |
| PLT level, ×10^9^/L | 174.00（137.00-219.00） | 177.00(149.00-259.00) | 166.00(127.00-214.00) | 212.00(158.00-268.00) | 175.00(143.00-219.00) | 0.074 |
| RBC level, ×10/L | 3.91（3.52-4.29） | 3.66(3.09-4.02) | 4.07(3.61-4.45) | 3.91(2.97-4.33) | 3.91(3.58-4.25) | 0.037 |
| Hematological indicators before discharge | | | | | | |
| RBC level, ×10^12^/L | 3.83（3.44-4.13） | 3.52(3.05-3.92) | 3.90(3.50-4.22) | 3.81(3.33-4.14) | 3.90(3.45-4.19) | 0.025 |
| PLT level, ×10^9^/L | 228.00（187.00-289.00） | 224.00(189.00-324.00) | 228.00(185.00-282.00) | 267.50(227.75-306.50) | 221.00(186.00-282.00) | 0.412 |
| ALT, U/L | 32.00（22.00-52.00） | 23.00(17.00-37.00) | 34.00(23.00-52.00) | 29.00(21.00-50.75) | 34.00(23.00-58.00) | 0.090 |
| AST, U/L | 28.00（22.00-37.00） | 27.00(20.00-37.00) | 30.00(23.00-38.00) | 24.50(21.00-40.75) | 29.00(20.00-35.00) | 0.725 |
| INR | 1.06（1.01-1.12） | 1.07(0.99-1.13) | 1.06(1.01-1.15) | 1.03(1.00-1.08) | 1.06(1.01-1.11) | 0.451 |
| PT, s | 13.80（13.30-14.30） | 13.80(13.20-14.30) | 13.90(13.30-14.50) | 13.50(13.10-14.03) | 13.80(13.30-14.30) | 0.551 |
| WBC level, ×10^9^/L | 6.29(4.96-7.69) | 6.29(4.63-7.48) | 6.36(5.23-7.61) | 4.98(4.16-6.98) | 6.69(5.06-8.11) | 0.167 |

**Table_S3.** Logistic Regression for Predictive Factors of Postoperative Complications

|  | **Univariate analysis** | | **Multivariate analysis** | |
| --- | --- | --- | --- | --- |
|  | **HR (95% CI)** | **P value** | **HR (95% CI)** | **P value** |
| Age (years) | 1.31 (1.09-1.58) | 0.415 |  |  |
| Sex | 1.32 (0.68-9.44) | 0.681 |  |  |
| ASA grade |  |  |  |  |
| I |  | 1.000 |  |  |
| II | 0.39（0.28-2.54） | 0.532 |  |  |
| III | 1.66（0.69-6.28） | 0.731 |  |  |
| Open approach | **1.89 (0.93-4.42)** | **0.035** | **2.56 (1.01-6.49)** | **0.004** |
| Major hepatectomy | 2.34 (1.13-4.56) | 0.493 |  |  |
| Operative time (min) | 1.00（0.99-1.01） | 0.936 |  |  |
| Blood loss >400 (ml) | 1.43（0.36-5.70） | 0.716 |  |  |
| Normal muscle mass  and strength |  | 1.000 |  |  |
| Low muscle mass and strength | **4.92 (1.77-69.93)** | **<0.001** | **4.21 (1.44-9.48)** | **0.025** |
| Low muscle mass but normal muscle strength | **1.23 (1.09-6.35)** | **0.002** | 0.61 (0.22-1.69) | 0.339 |
| Low muscle strength but normal muscle mass | **1.31 (1.19-9.44)** | **0.022** | 1.19 (0.31-4.56) | 0.803 |
| Body Mass Index (kg/m²) | 0.99 (0.92-1.11) | 0.640 |  |  |
| Abdominal circumference (cm) | 1.02 (0.98-1.07) | 0.935 |  |  |
| Visceral adipose tissue (cm²) | 1.00 (1.00-1.00) | 0.423 |  |  |
| Subcutaneous adipose tissue (cm²) | 1.00 (1.00-1.00) | 0.241 |  |  |
| Skeletal Muscle Index (cm/m²) | 1.03 (0.95-1.13) | 0.555 |  |  |
| Handgrip strength (kg) | 0.99 (0.90-1.09) | 0.422 |  |  |
| The chair stand test (s) | 1.08 (0.97-1.21) | 0.192 |  |  |
| Gait speed (m/s) | 0.99 (0.79-1.32) | 0.260 |  |  |
| Physical activity >= 4h/wk | 1.98 (0.74-5.29) | 0.260 |  |  |
| Age-adjusted Charlson comorbidity score | 1.05（0.79-1.57） | 0.743 |  |  |
| Child-Pugh stage B | 1.02（0.42-3.21） | 0.482 |  |  |
| MELD score | 1.42（0.99-2.03） | 0.115 |  |  |
| BCLC stage |  |  |  |  |
| 0 |  | 1.000 |  |  |
| A | 1.03（0.22-4.04） | 0.992 |  |  |
| B | 1.95（0.19-4.32） | 0.537 |  |  |
| C | 2.53（0.26-4.43） | 0.631 |  |  |
| Semi-vegetarian |  | 1.000 |  |  |
| Meat eater | 0.96 (0.31-3.02) | 0.949 |  |  |
| Vegetarian | 0.19 (0.02-1.76) | 0.142 |  |  |
| Sleep time > 8h | 0.87 (0.34-2.20) | 0.762 |  |  |
| Smoking | 0.98 (0.24-4.09) | 0.980 |  |  |
| Drinking | 1.26 (0.68-3.06) | 0.607 |  |  |
| Hypertension | 3.85 (0.98-8.31) | 0.881 |  |  |
| Diabetes | 0.62 (0.11-1.42) | 0.348 |  |  |
| Hematological indicators of the first admission | | | | |
| AST (U/L) | 1.08 (1.02-1.15) | 0.061 |  |  |
| ALT (U/L) | 0.95 (0.91-1.00) | 0.172 |  |  |
| GGT level (U/L) | 1.00 (1.00-1.00) | 0.224 |  |  |
| Cholesterol (mmol/L) | 1.00 (0.11-5.84) | 0.390 |  |  |
| TBIL (Umol/L) | 1.01 (1.00-1.03) | 0.196 |  |  |
| Cr (Umol/L) | 1.01 (0.99-1.04) | 0.477 |  |  |
| ALB (g/L) | 1.09 (0.91-1.30) | 0.379 |  |  |
| TG (mmol/L) | **0.06 (0.05-0.72)** | **0.026** | 0.80 (0.51-1.26) | 0.328 |
| HDL (mmol/L) | 0.73 (0.03-2.68) | 0.276 |  |  |
| LDL (mmol/L) | 0.95 (0.22-2.49) | 0.353 |  |  |
| LDH (U/L) | 1.00 (0.99-1.00) | 0.182 |  |  |
| INR | 1.00 (1.00-1.00) | 0.055 |  |  |
| PT (s) | 6.11 (0.47-79.39) | 0.062 |  |  |
| AFP>=400 (ng/Ml) | 1.00 (1.00-1.00) | 0.219 |  |  |
| CEA (Ug/L) | 1.10 (1.00-1.22) | 0.238 |  |  |
| CA199 (U/mL) | 1.00 (1.00-1.00) | 0.326 |  |  |
| WBC level (10^9^/L) | 2.37 (0.38-12.76) | 0.366 |  |  |
| RBC level (10^12^/L) | 0.67 (0.26-2.83) | 0.369 |  |  |
| NEUT level (10^9^/L) | 0.46 (0.20-1.07) | 0.074 |  |  |
| Monocyte level (10^9^/L) | 1.00 (0.99-1.01) | 0.310 |  |  |
| HGB level (g/L) | 0.98 (0.94-1.03) | 0.328 |  |  |
| PLT count (10^9^/L) | 1.00 (1.00-1.01) | 0.112 |  |  |
| Fib level (g/L) | 1.42 (0.68-2.96) | 0.159 |  |  |

Abbreviations: ALT, alanine aminotransferase; AST, aspartate aminotransferase; GGT, γ-glutamyltransferase; TBIL, total bilirubin; Cr, creatinine; ALB, albumin; TG, triglyceride; HDL, high density lipoprotein; LDL, low density lipoprotein; LDH, Lactate dehydrogenase; INR, international normalized ratio; PT, Prothrombin time; AFP, alpha-fetoprotein; CEA, carcinoembryonic antigen; CA199, Saccharide antigen 199; WBC, white blood cell; RBC, red blood cell; NEUT, neutrophil; HGB, hemoglobin;PLT, blood platelet; Fib, Fibrinogen.
